# Psychosocial Effects of the COVID-19 Pandemic: Large-scale Quasi-Experimental Study on Social Media

**DOI:** 10.2196/22600

**Published:** 2020-11-24

**Authors:** Koustuv Saha, John Torous, Eric D Caine, Munmun De Choudhury

**Affiliations:** 1 School of Interactive Computing Georgia Institute of Technology Atlanta, GA United States; 2 Division of Digital Psychiatry Beth Israel Deaconess Medical Center Harvard Medical School Boston, MA United States; 3 Department of Psychiatry University of Rochester Rochester, NY United States

**Keywords:** social media, Twitter, language, psychosocial effects, mental health, transfer learning, depression, anxiety, stress, social support, emotions, COVID-19, coronavirus, crisis

## Abstract

**Background:**

The COVID-19 pandemic has caused several disruptions in personal and collective lives worldwide. The uncertainties surrounding the pandemic have also led to multifaceted mental health concerns, which can be exacerbated with precautionary measures such as social distancing and self-quarantining, as well as societal impacts such as economic downturn and job loss. Despite noting this as a “mental health tsunami”, the psychological effects of the COVID-19 crisis remain unexplored at scale. Consequently, public health stakeholders are currently limited in identifying ways to provide timely and tailored support during these circumstances.

**Objective:**

Our study aims to provide insights regarding people’s psychosocial concerns during the COVID-19 pandemic by leveraging social media data. We aim to study the temporal and linguistic changes in symptomatic mental health and support expressions in the pandemic context.

**Methods:**

We obtained about 60 million Twitter streaming posts originating from the United States from March 24 to May 24, 2020, and compared these with about 40 million posts from a comparable period in 2019 to attribute the effect of COVID-19 on people’s social media self-disclosure. Using these data sets, we studied people’s self-disclosure on social media in terms of symptomatic mental health concerns and expressions of support. We employed transfer learning classifiers that identified the social media language indicative of mental health outcomes (anxiety, depression, stress, and suicidal ideation) and support (emotional and informational support). We then examined the changes in psychosocial expressions over time and language, comparing the 2020 and 2019 data sets.

**Results:**

We found that all of the examined psychosocial expressions have significantly increased during the COVID-19 crisis—mental health symptomatic expressions have increased by about 14%, and support expressions have increased by about 5%, both thematically related to COVID-19. We also observed a steady decline and eventual plateauing in these expressions during the COVID-19 pandemic, which may have been due to habituation or due to supportive policy measures enacted during this period. Our language analyses highlighted that people express concerns that are specific to and contextually related to the COVID-19 crisis.

**Conclusions:**

We studied the psychosocial effects of the COVID-19 crisis by using social media data from 2020, finding that people’s mental health symptomatic and support expressions significantly increased during the COVID-19 period as compared to similar data from 2019. However, this effect gradually lessened over time, suggesting that people adapted to the circumstances and their “new normal.” Our linguistic analyses revealed that people expressed mental health concerns regarding personal and professional challenges, health care and precautionary measures, and pandemic-related awareness. This study shows the potential to provide insights to mental health care and stakeholders and policy makers in planning and implementing measures to mitigate mental health risks amid the health crisis.

## Introduction

The impacts of global public health emergencies extend beyond medical repercussions; they affect individuals and societies on many levels, causing disruptions [1,2]. In an article written by the American Psychological Association following the Ebola outbreak [3], the epidemic was described as an “epidemic of fear.” In the United States, it was labeled by the media as “fearbola” to describe a paranoia that infected the country. Reports of similar feelings of anxiety, stress, and uncertainty have been repeatedly reported during other global outbreaks and pandemics [4]. The ongoing outbreak of SARS-COV-2 has led to a pandemic of illness (COVID-19) that has caused 16 million cases and 700,000 deaths worldwide, reported as of the end of July 2020 [5]. According to recent surveys from the Census Bureau, the Centers for Disease Control and Prevention, and other studies, the COVID-19 crisis has been reported to be associated with rapid rises in psychological distress across many nations [6], with women, the young, the less educated, and some ethnic minority groups reporting greater mental health strain [7]. On the one hand, people are worried about the direct effects of potential infection, including fears of death, lasting disabilities, or exacerbating chronic illnesses. On the other hand, actions to mitigate the spread of COVID-19, including social distancing, quarantines, and business closures with resulting job losses, are a source of life disruptions and emotional distress.

Fear and anxiety about a disease as infectious as COVID-19 can trigger new-onset or exacerbate existing mental illness [8]. Therefore, the practical impact of the crisis is far greater than the actual number of infection cases or fatalities [8,9]. Although expressions of distress may stem from concern and worry relating to the direct impacts of the disease, they may relate as much to disruption of regular routines, sleep and eating patterns, having children out of school and at home full-time, economic hardships and unusual volatility in financial markets, and forced geographical displacement or confinement. Indeed, some people are at risk of developing posttraumatic distress due to exposure to the multifaceted uncertainties or from confronting dying people or lost loved ones. Although disease mitigating efforts such as “social distancing” and “self-quarantining” are recommended [10-12], individuals in medical isolation may experience increased symptoms of anxiety and depression, as well as feelings of fear, abandonment, loneliness, and stigmatization [13,14].

Despite concerns about the myriad of social and behavioral issues associated with the COVID-19 pandemic [15,16], there has been scant research to examine its psychosocial impacts or how to predict and mitigate them. Although it is anticipated that COVID-19 will have broadly ramifying effects [17,18], public health workers and crisis interventionists are limited in their ability to extend services and support in a timely, preemptive fashion. Although surveys are a step forward to support such efforts [7], due to their retrospective recall bias, limited scalability, and inability to provide real-time insights, public health workers are not only unable to prioritize services for the most vulnerable populations but also, more specifically, less equipped to direct prevention efforts toward individuals with greater propensities for adverse psychological impacts.

This paper seeks to address the aforementioned gap by drawing insights into people’s expressed mental health concerns by leveraging social media data. The rise in online and social media activity has provided an unprecedented opportunity to enhance the identification and monitoring strategies of various mental and psychosocial disorders [7,19]. Over 80% of US adults use social media daily [20], placing it ahead of texting, email, and instant messaging, and disclose considerably more about themselves online than offline [21,22]. Social media provides a real-time platform where people often candidly self-disclose their concerns, opinions, and struggles during this pandemic [23]. In particular, our study targets the following research aims:

- Aim 1: To quantitatively assess the psychosocial effects of the COVID-19 pandemic using social media data
- Aim 2: To examine how the psychosocial effects of the COVID-19 pandemic have varied over time
- Aim 3: To examine if social media language reflects the major psychosocial concerns during the COVID-19 pandemic

For these research questions, we measured psychosocial effects in terms of symptomatic mental health expressions of anxiety, depression, stress, and suicidal ideation, and expressions seeking emotional and informational support. Our study is founded on a large body of work on studying mental health and psychosocial dynamics with social media data [24-30]. Several studies have leveraged Twitter (which is also the data we used) to study health attributes and public health [30], including symptoms related to diseases [31], disease contagion [32], obesity and physical health [33], mood and depressive disorders [28,34], mental health self-disclosures [27], posttraumatic stress disorder [35], addictive behaviors and substance use [36,37], etc. Because social media data (and Twitter posts in particular) are recorded in the moment, they provide rich information about both the individual as well as the larger world [30]. In particular, we draw on two kinds of prior work: symptomatic mental health expressions and support expressions. Related to the former, Saha et al [25], in their study on the effects of psychiatric medications on Twitter, developed classifiers of mental health symptomatic expressions using social media language, which we replicated in this study. Related to the latter, we draw upon Sharma and De Choudhury’s [38], and Saha and Sharma’s [39] developed classifiers of social support expressions, specifically emotional and informational support.

## Methods

### Data

#### Using Twitter Data

To study people’s psychosocial expressions on social media, we obtained Twitter data. Twitter is one of the most popular social media platforms, and its public-facing, microblogging–based design enables candid self-disclosure and self-expressions for individuals [27].

#### Twitter Streaming Application Programming Interface

We collected data in our study using the Twitter Streaming application programming interface (API). The Twitter Streaming API is an official data collection API that Twitter shares with researchers providing free access to a 1% sample of its data on parameters set by researchers. That is, for a given set of parameters, Twitter queries the volume of available data at a particular moment [40]. If the volume of the query exceeds 1% of all Twitter posts at that moment, then the response is sampled to be less than 1%. However, the Twitter Streaming API is like a black-box with a lack of transparency in the sampling methodology [41,42], yet this is one of the few forms of unfettered and large-scale social media data access to researchers outside social media companies [43] and has been used in prior research, including in health-related studies [35,44,45]. The Discussion section revisits the limitations of our study due to the challenges of the Twitter Streaming API.

For the purposes of our study, we used two kinds of parameters: (1) language of a Twitter post as “english” and (2) geolocation bounds set to be within the geographic coordinates of the United States. Therefore, our following analyses concern Twitter data that at least fulfill both these criteria. We note that the location filter additionally prevents any retweets in the data set, as retweets are not geolocation labeled by design on Twitter [40], allowing us to study only originally created Twitter posts.

#### Treatment Data

In particular, we focused our study on the US population and leveraged the Twitter Streaming API. Using geo-bounded coordinates, we collected 1% of real-time Twitter data originating from the United States. We collected 59,096,694 Twitter posts between March 24, 2020, and May 24, 2020. Because this data set comes from the same period when the COVID-19 outbreak occurred, we labeled this data set as the *treatment* data set. We note that this period saw an exponential growth in reported COVID-19 infection cases (about 50,000-1 million) and fatalities (about 1000-56,000) in the United States [46]. During these 2 months, federal and state policies and laws were enacted to control or mitigate the spread of the outbreak, including school and work closures; stay-at-home orders; and the Coronavirus Aid, Relief, and Economic Security Act [47].

#### Control Data

To understand the social media expressions particularly attributed to the COVID-19 crisis, we obtained a control data set that originated from the same geographical location (the United States) and a similar time period but from the previous year (2019). Prior work [47,48] motivated this approach of obtaining control data that acts as a baseline and likely minimizes confounding effects due to geo-temporal seasonality in lifestyle, activities, experiences, and unrelated events that may have some psychosocial bearing. We obtained a similarly sized data set of 40,875,185 Twitter posts shared between March 24, 2019, and May 24, 2019.

Both the treatment and control data sets were collected in real time, and therefore, they were the entire 1% sample of Twitter posts returned in real time; we did not conduct any additional sampling on this data. We note that the size of the control data was smaller than that of the treatment [42] despite each consisting of the same 2-month duration. This could be because the volume of posts [40,42] on Twitter increased significantly in 2020 [49], leading to an increase in the 1% sample as well. However, we cannot make any such conclusion, especially because of the lack of transparency in how Twitter conducts the 1% sampling [42].

### Psychosocial Effects of COVID-19

#### Study Design

Toward our first research aim of understanding the psychosocial impacts of the COVID-19 outbreak, we conducted two types of analysis on our Twitter data set, which we describe in the following sections. Our study builds upon the vast, rapidly growing literature studying mental health concerns and psychosocial expressions within social media data [19,21,24-28,34,48,50-52]. We adopted a quasi-experimental study design, which minimizes for geo-temporal confounds by using Twitter data sets from similar regions and similar times of the year in a treatment (2020) and a control (2019) year.

#### Symptomatic Mental Health Expressions

Drawing on the work previously referenced, we hypothesize that people’s self-disclosure expressions on social media can reveal symptomatic mental health expressions attributed to the COVID-19 crisis. We examined symptomatic expressions of anxiety, depression, stress, and suicidal ideation. These are not only some of the most critical mental health concerns but also have been attributed to be consequences of the pandemic outbreak [15,53,54].

To identify mental health symptomatic expressions in social media language, Saha et al [25] built machine learning classifiers using transfer learning methodologies—the main idea here is to infer mental health attributes in an unlabeled data by transferring a classifier trained on a different labeled data set. These classifiers are *n*-gram–based (*n*=1,2,3) binary support vector machine (SVM) models, where the positive class of the training data sets stems from appropriate Reddit communities (*r/depression* for depression, *r/anxiety* for anxiety, *r/stress* for stress, and *r/SuicideWatch* for suicidal ideation), and the negative class of training data sets comes from nonmental health–related content on Reddit—a collated sample of 20 million posts gathered from 20 subreddits from the landing page of Reddit during the same period as the mental health subreddit posts, such as *r/AskReddit*, *r/aww*, and *r/movies*. These classifiers perform at a high accuracy of approximately 0.90 on average on held-out test data [25].

##### Clinical Validity

Saha et al’s [25] classifiers used here have also been shown to transfer well on Twitter with an 87% agreement between machine-predicted labels and expert appraisal [48], where experts annotated posts in the classification test data using Diagnostic and Statistical Manual of Mental Disorders, 5th Edition [55] criteria of mental health symptoms. Bagroy et al [56] reported additional validation of such derived insights with feedback from clinical experts [55]. In this study, the outcomes of the mental health expression classifiers were compared with those given by human coders on the same (random) sample of social media posts; the latter coded the posts based on a codebook developed using prior qualitative and quantitative studies of mental health disclosures on social media and literature in psychology on markers of mental health expressions. Coders not only agreed with the outcomes of the classifiers (κ=0.83) but also noted that the classifiers could identify explicit expressions of firsthand experience of psychological distress or mental health concerns (“i get overwhelmingly depressed”) as well as expressions of support, help, or advice around difficult life challenges and experiences (“are there any resources I can use to talk to someone about depression?”). Further details about these classifiers, including their detailed performance, predictive features demonstrating model interpretability, and efficacy of transfer to Twitter data, can be found in [25,48,56]. We used these classifiers to machine label both our *treatment* and *control* data sets. Textbox 1 shows example Twitter posts in our data set that exhibit mental health symptomatic expressions (because many of these labels were comorbid, we show example posts that exhibit one or more of these mental health symptomatic expressions).

**Example paraphrased posts in the treatment data that exhibited high symptomatic mental health expressions.**

- I am so sick and tired of the #coronavirus (anxiety, stress)
- The kind of person I am, I don’t deserve to meet these people (depression)
- 2020 is the saddest year. There is a lack of money, necessities needed for daily life are gone from stores! I am at work as a healthcare professional hurting for my patients because they can’t see their family. (anxiety, stress)
- I am too overwhelmed by school and having a crippling anxiety to keep up with everything online I’m seriously NOT OK! I CAN’T TAKE THIS! (anxiety, suicidal ideation)
- During the online lecture, the prof. asked each of us how we were doing and feeling, I said, I am too anxious to know what’s next, and i keep thinking about what’s gonna happen, and she was like okay chill! (depression, anxiety, stress)

#### Support Expressions

Social support is considered an essential component in helping people cope with psychological distress [57]. Research reports that supportive interactions can even have a “buffering effect” [57,58]; that is, they can be protective against the negative consequences of mental health. With the wide adoption of web and social media technologies, support seeking (and providing) is increasingly happening online and has been shown to be efficacious [21,59]. In fact, a meta-analysis indicated that online support is effective in decreasing depression and increasing self-efficacy and quality of life [60]. In the context of suicide, certain types of social support in Reddit communities may reduce the chances of future suicidal ideation among those seeking mental health help [61]. Oh et al [62] further showed that surveyed Facebook users demonstrated a positive relationship between having health concerns and seeking health-related social support. Indeed, during global crises such as COVID-19, when many of the physical sites for health care (including mental health) have been closed or have restricted access, it is likely that online support has proliferated [63]. Fear of potential infection may further have alienated individuals in need to pursue formal treatment, therapy, and support, perhaps channelizing their support-seeking efforts online and on social media.

According to the “Social Support Behavioral Code” [64], two forms of support that have received theoretical and empirical attention are emotional and informational support. Emotional support (ES) corresponds to empathy, encouragement, and kindness, while informational support (IS) corresponds to information, guidance, and suggestions [38,65]. These two forms of support have been found to be most prevalent and effective in several studies of online support and social media [38,62,66,67]. Social media enables individuals to self-disclose and express their emotional and informational needs [67]. Andalibi et al [66] found that these two kinds of support can co-occur with other forms of support, such as posts seeking ES often seeking esteem and network support, and Attai et al [68] noted that Twitter is effective in seeking and providing health-related informational needs, contextually related with our problem of interest.

To identify support expressions on social media, we used an expert-appraised data set and classifier built in prior studies [38,39]. These are binary SVM classifiers identifying the degree (high and low) of ES and IS in social media posts. When the predictions of these classifiers were cross-validated with expert annotations from Sharma and De Choudhury’s [38] data, the classifiers were found to have *k*-fold cross-validation accuracies of 0.71 and 0.77 in ES and IS classifications, respectively [39]. Similar to the symptomatic expressions classifiers, the classifiers of support expressions are transferred from Reddit and typically performed well in our data set due to the high linguistic equivalence between Reddit and Twitter data sets [34]. We further manually inspected a random set of 125 Twitter posts in our data set using the methods outlined in prior studies [25,56] to rate each Twitter post with binary high or low ES and IS. We found that the manual ratings and classifier ratings showed a high agreement of 88% and 93%, respectively, indicating statistically significant transfer classification on Twitter. We used these classifiers to label the presence of ES and IS in our *treatment* and *control* data sets. Textbox 2 shows a few example paraphrased posts of support expressions in our *treatment* data set.

**Example paraphrased posts in the treatment data on support expressions.**

- To our residents, the town is here for you but we need your help if we are going to keep our hospitals from being overwhelmed. (emotional and informational support)
- I hope you are safe and healthy! Keep the faith God never fails us & always has perfect plan. (emotional support)
- my wife is laid off. She has been trying for days to get ahold of unemployment. Please help. We need income. Running low on basic things. (emotional and informational support)
- Dear God, we are going through some struggles these days. Could you please send us some sunshine? Thanks so much. (emotional support)
- According to my mom, kindness is needed more than ever now. So, send love to you! How are you being kind to others today? (emotional support)

### Examining Psychosocial Expressions Over Time and Language

#### Approach Overview

Next, we describe the methods to examine how the COVID-19 pandemic may have caused changes in psychological expressions by comparing our *treatment* (outbreak year) and *control* (no outbreak year) data sets. For both our data sets, we aggregated the number of posts that expressed symptomatic and support expressions by day and by type. We compared the pervasiveness of each kind of measure in the data sets along with conducting statistical significance in their differences using two-sample *t* tests and effect sizes (Cohen *d*).

#### Temporal Variation

For our second research aim, we compared the daily variation of measures between the *treatment* and *control* data sets, we transformed our data into standardized *z* scores. Our data sets relied on the Twitter Streaming API and were subject to daily inconsistencies of available data each day [41]. Transformed *z* scores are not sensitive to such absolute values and inconsistencies, and essentially quantify the number of SDs by which the value of the raw score is above or below the mean. Similar standardization techniques have been adopted in prior social media time series studies [48,69]. *Z* scores were calculated as *(x − μ) / σ*, where *x* is the raw value, *μ* is the mean, and σ is the SD of the population. Here, to obtain population *μ* and *σ*, in addition to our *treatment* and *control* data, we also included a year-long Twitter data of over 240 million Twitter posts (September 2018 to August 2019). For each of the measures in symptomatic and support expressions, we first calculated *μ* and *σ* on the per-day occurrence of that particular measure in the data set of over 300 million Twitter posts (combining 240 million posts between September 2018 and August 2019, and 60 million posts in the treatment data between March and May 2020). For each measure, we then calculated the *z* score per day and interpreted the positive *z* scores as values above the mean and negative *z* scores as those below the mean.

#### Linguistic Differences

For our third research aim, we examined COVID-19–related linguistic differences in the psychosocial expressions on social media, we employed an unsupervised language modeling technique, the Sparse Additive Generative Model (SAGE) [70]. Given any two data sets, SAGE selects salient keywords by comparing the parameters of two logistically parameterized-multinomial models using a self-tuned regularization parameter to control the trade-off between frequent and rare keywords. We conducted SAGE to identify distinguishing *n*-grams (*n*=1,2,3) between the *treatment* and *control* data sets, where each *n*-gram was returned with a SAGE score. The magnitude of an *n*-gram’s SAGE score signals the degree of its “uniqueness” or saliency, and in our case, a positive SAGE score (above 0) indicated that the *n*-gram was more salient in the *treatment* data, whereas a negative SAGE score (below 0) denoted greater saliency in the *control* data.

SAGE allowed us to obtain how the expressions differ during the COVID-19 outbreak as compared to the *control* period. We conducted two SAGE analyses, one each for symptomatic and support expressions. For the symptomatic expressions, we first obtained posts that were indicative of either anxiety, depression, stress, or suicidal ideation in the *treatment* and *control* data sets, and obtained SAGE for both. We used a similar method for support expressions by obtaining posts that were indicative of either emotional or informational support.

Finally, we cross-examined the salient keywords across symptomatic and support expressions to study how concerns were prevalent in either or both of the expression types. We measured log-likelihood ratios (LLRs) along with add-one smoothing, where LLRs close to 0 indicated comparable frequencies, LLRs<1 indicated the greater frequency in symptomatic expressions, and LLRs>1 indicated the greater frequency in support expressions. Together, these linguistic analyses enabled us to obtain psychological concerns and understand how COVID-19 has psychosocially affected individuals, and to contextualize these concerns in the literature on consequences of global crises.

## Results

### Summary of Results

We summarize our first set of results in Table 1. For all our measures, we found statistical significance (as per *t* tests) as well as significant effect sizes (Cohen *d*>0.4 for all measures [71]) in social media expressions in the *treatment* data as compared to that in the *control* data. Assuming that most other confounders were minimized due to the geo-temporal similarity of the data sets, our findings indicated that the COVID-19 outbreak led to an increase in people’s symptomatic and support expressions of mental health. We elaborate on the results in the following sections.

**Table 1:** Comparing social media expressions in the treatment (2020) and control (2019) data sets.

Expression		Treatment (2020), mean (SD)	Control (2019), mean (SD)	Δ (%)	Cohen *d*	*t* test (*df*)	*P* value
**Symptomatic mental health expressions**							
	Anxiety	1.65 (0.20)	1.35 (0.08)	21.32	1.96	12.60 (151)	<.001
	Depression	9.00 (0.60)	8.17 (0.35)	10.18	1.71	10.72 (151)	<.001
	Stress	19.31 (0.77)	18.61 (0.43)	3.76	0.81	3.65 (151)	.009
	Suicidal ideation	3.14 (0.31)	2.62 (0.13)	19.73	2.14	13.54 (151)	<.001
**Support expressions**							
	Emotional support	8.56 (0.84)	8.17 (0.50)	4.77	0.46	2.87 (151)	.004
	Informational support	1.75 (0.18)	1.67 (0.08)	4.78	0.56	3.58 (151)	<.001

### Temporal Variation

**Figure 1:**
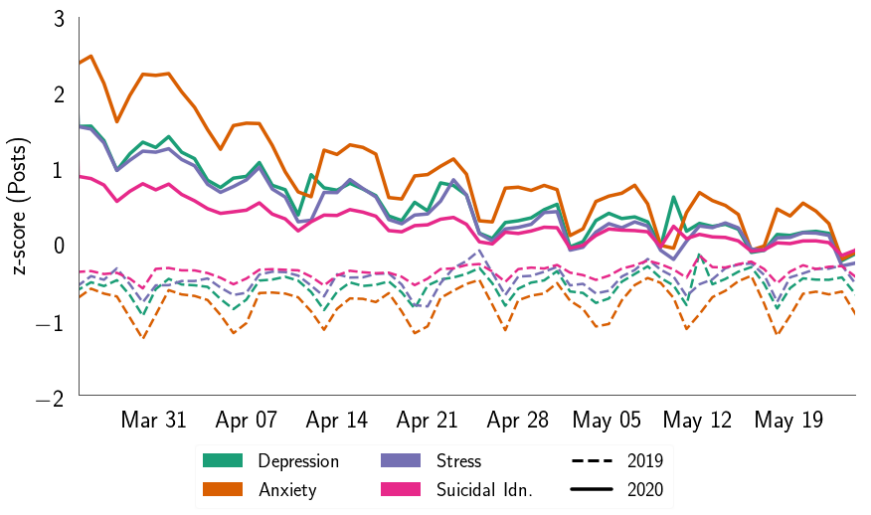
Comparison of symptomatic mental health expressions on social media posts in the same period (March 24 to May 24) in 2019 and 2020 (COVID-19 outbreak year). Idn.: ideation.

**Figure 2:**
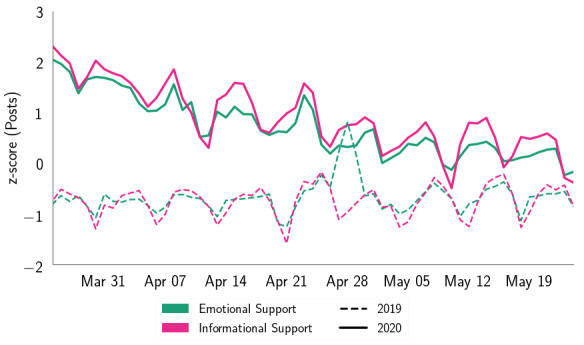
Comparison of support expressions on social media posts in the same period (March 24 to May 24) in 2019 and 2020 (COVID-19 outbreak year).

Figure 1 shows the changes in symptomatic mental health expressions for the same period in the *treatment* (2020) and *control* (2019) years. We found that the *treatment* and *control* data sets showed significant differences in people’s symptomatic expressions (Table 1), among which anxiety showed the most significant increase (21.32%), followed by suicidal ideation (19.73%), depression (10.18%), and stress (3.76%). Figure 2 shows the evolution of support expressions change in the *treatment* and *control* data sets. The differences were significant (Table 1), and we found that ES increased by 4.77% and IS increased by 4.78%.

In both the plots of Figures 1 and 2, we found a general trend of negative slope (average slope=–0.03) within the *treatment* year, which was closer to zero slope (average slope=3.19*10^−4^) in the *control* data set. This may suggest that within the *treatment* year, people’s mental health expressions gradually leveled out over time, despite the growing rate of COVID-19 active cases. The plots indicated that psychological expressions almost converge at the tails. This could likely be due to people’s habituation with the situation and surroundings with the passage of time [70,72], as has been observed for other crisis events [48,73]; however, this needs to be explored further. Within the *control* data set, we observed a sudden peak on April 28, 2019, which could be attributed to a shooting incident at a synagogue in San Diego [74]. The observations reflected that the COVID-19 pandemic has increased people’s mental health expressions on social media, aligning with other contemporary literature and media reports [8,53].

### Linguistic Expressions

#### Symptomatic Mental Health Expressions

Table 2 summarizes the language differences as per SAGE for posts expressing high mental health expressions in the *treatment* and *control* periods—keywords with positive SAGE saliently occurred in the *treatment* data, whereas those with negative SAGE saliently occurred in the *control* data. A majority of the keywords that occurred in the *treatment* period were contextually related to the COVID-19 pandemic, such as *covid19*, *coronavirus*, *social distancing*, and *stayathome isolation*. These keywords were used in posts expressing mental health concerns either explicitly (eg, “Social distancing is both sad and anxiety-inducing at the same moment”) or implicitly (eg, “In order to get my family treated, I will do more than beg, and I will donate 25K for research to develop COVID19 vaccine”). We also found that the *treatment* period used keywords referring to key personnel such as *dr fauci* (referring to Anthony Fauci, one of the leads in the incumbent White House Coronavirus Task Force in the United States and Director of the National Institute of Allergy and Infectious Diseases since 1984 [75]) and political figures like *Nury Martinez* and *Donald Trump*. Further, we found keywords such as *essential workers*, *doctor jobs*, and *risking lives*, which describe high-risk worker situations (eg, “I am not complaining about going to work, rather, I am concerned about risking my health for work”), and certain treatment suggestions that evolved during this period [76], such as *garlic*, *malaria*, and *hydroxychloroquine* (eg, “I do worry tho! He is focused on job numbers, approval? ratings and repeating mistruths. His spouting of 2 drugs, one for malaria & the other a Z-pack. A Senior couple tried these untested drugs; wife is in ICU & husband also hospitalized! This is irresponsible & dangerous!”).

**Table 2:** Top salient n-grams (n=1,2,3) for symptomatic mental health concerns in the treatment and control data sets (SAGE analysis [70]).

Salient in treatment (2020)		Salient in control (2019)	
Keyword	SAGE^a,b^	Keyword	SAGE^c^
covid19	11.17	hospitality	–2.81
lord marvelous	10.87	trainee	–2.78
coronavirus	10.58	crimes	–2.74
social distancing	9.92	delay	–2.55
nury martinez	9.66	traffic	–2.55
working councilwoman	9.66	accident	–2.39
bored daily	8.69	finance accounting	–2.26
stayathome isolation	8.69	half finance	–2.22
quarantinelife	8.62	auburn	–2.21
quarantine got	7.87	half technology	–2.19
securityguard	7.63	pete	–2.19
essential workers	7.62	parttime	–2.18
dr fauci	7.56	robert half	–2.12
went tired	7.48	tickets	–2.08
coronaviruspandemic	7.44	marvel	–2.07
flattenthecurve	7.44	tournament	–1.92
doctorjobs	7.32	muslim	–1.90
garlic	7.29	florida	–1.90
hydroxychloroquine	7.28	boston	–1.88
n95 masks	7.26	cashier	–1.87
masks gloves	7.26	playoffs	–1.86
practice social distancing	7.21	sales representative	–1.85
physicianjobs doctorjobs	7.13	springfield	–1.84
quarantine life	6.98	border	–1.84
plz help small	6.96	barista	–1.77
small donation	6.96	israel	–1.77
stay home orders	6.92	nc click	–1.76
self quarantine	6.88	playoff	–1.75
positive covid19	6.79	bracket	–1.75
risking lives	6.79	terrorist	–1.65

^a^SAGE: Sparse Additive Generative Model.

^b^Positive SAGE scores indicated greater saliency in the treatment (2020) data.

^c^Negative SAGE scores indicated greater saliency in the control (2019) data.

#### Support Expressions

Table 3 lists the top keywords as per SAGE for support posts in the *treatment* and *control* periods. Keywords with positive SAGE saliently occurred in the *treatment* data, whereas those with negative SAGE saliently occurred in the *control* data. We found keywords that explicitly relate to COVID-19 occurred in the *treatment* period. We also found that the *treatment* period consisted of posts that seek support related to job and pay, such as *losing jobs*, *need pay*, and *furloughed* (eg, “Many in our community have lost their jobs, are underinsured and are struggling to make ends meet. Providing pantries, hot meals, hotspots and distance learning opportunities is now more critical than ever, please donate”). Our data also revealed the prevalence of contextually related keywords such as *masks*, *ppe*, *hoarding*, *stockpile*, and *sanitizer* that are medically recommended prevention and containment measures of COVID-19 infection (eg, “Please contact me if you have any N95 mask or know to obtain some. My sister and a few friends work in the OR and they do not have the supplies to stay safe, they have patients who have #COVID19. TY! #HealthcareHeroes”).

**Table 3:** Top salient n-grams (n=1,2,3) for support expressions in treatment and control data sets (SAGE analysis [70]).

Salient in treatment (2020)		Salient in control (2019)	
Keyword	SAGE^a,b^	Keyword	SAGE^c^
lord	7.93	hospitality	–2.86
fauci	6.70	duke	–2.51
ventilators	6.59	shift supervisor	–2.24
quarantine	6.47	tampa	–2.21
securityofficer	6.11	advisor	–1.95
n95	5.53	customerservice	–1.92
hope staying safe	5.36	investigation	–1.89
ppe	5.25	manager retail	–1.87
wearing masks	5.20	traffic	–1.87
uncertain times	5.16	muslim	–1.86
healthcare workers	5.01	store manager	–1.85
furloughed	5.00	tickets	–1.85
asymptomatic	4.95	playoffs	–1.83
people quarantine	4.90	cubs	–1.82
fighting stigma	4.82	border	–1.81
staysafe	4.67	cashier	–1.79
food bills	4.66	springfield	–1.79
disinfectant	4.64	delay	–1.76
hand sanitizer	3.08	barista store	–1.76
clorox	3.03	boston	–1.76
medical supplies	2.97	counter	–1.75
trying times	2.89	barista	–1.74
risking lives	2.87	columbia	–1.73
stockpile	2.86	meeting retail	–1.73
father passed	2.36	informational meeting	–1.73
hoarding	2.31	stlouis	–1.72
mask	2.31	marvel	–1.70
medical professionals	2.27	marketing	–1.68
losing jobs	2.27	server	–1.67
toilet paper	2.05	accident	–1.64

^a^SAGE: Sparse Additive Generative Model.

^b^Positive SAGE scores indicated greater saliency in the treatment (2020) data.

^c^Negative SAGE scores indicated greater saliency in the control (2019) data.

#### Linguistic Comparability

Finally, Table 4 shows the results of the lexical comparability analysis, where LLRs demarcate the top keywords used for symptomatic mental health expressions and support expressions within the *treatment* data set. We found that keywords, such as safety precautions (*wear masks*), health care and treatment (*health care workers, hospitalized, beds,* and *icu*), and life and death (*passed away, kill people, human lives,* and *deaths*), comparably overlapped in both kinds of psychological expressions (LLRs~0). These keywords were also used to raise awareness and express solidarity with health care and high-risk workers (eg, “Taking all safety precautions and adhering to the guidelines established by our health care professionals will keep us safe”). Our lexico-psychological analyses revealed that more clinically relevant keywords and symptoms occurred frequently in symptomatic expressions (LLRs>0; eg, *sleep schedule* and *tested positive*), whereas socially relevant and stressful circumstances were more prevalent in support expressions (LLRs<0; eg, *im single parent, starve,* and *lost jobs*).

**Table 4:** Distribution of social media keywords across high symptomatic mental health and support expressions within the treatment period using LLRs.

LLRs^a^>0^b^		LLRs~0^c^		LLRs<0^d^	
Keyword	LLR	Keyword	LLR	Keyword	LLR
sleep schedule	0.75	infected	–0.01	im single parent	–1
lonely	0.64	wear masks	–0.01	starve	–1
anxiety	0.62	need help	–0.01	meditate	–1
isolation	0.56	kill people	–0.01	sorry loss	–0.73
stay safe	0.56	need pay	0	care people	–0.7
bored	0.56	health care workers	0	hard times	–0.45
tested positive	0.52	passed away	0	people sick	–0.4
quarantine life	0.52	seriousness	0	helping people	–0.4
homeschooling	0.51	human lives	0	sorry hear	–0.39
tired	0.5	deaths	0	urged	–0.33
doctor	0.48	domestic violence	0	new yorkers	–0.29
fighting stigma	0.46	comforting	0	lost jobs	–0.21
depression	0.45	hospitalized	0	hope family	–0.21
stuck inside	0.42	beds	0	selfish	–0.21
sane	0.41	icu	0.01	desperate	–0.21

^a^LLR: log likelihood ratio.

^b^Keywords with LLRs>0 distinctly occurred in high symptomatic expressions.

^c^Keywords with LLRs~0 occurred comparably in both symptomatic and support expressions.

^d^Keywords with LLRs<0 distinctly occurred in support expressions.

## Discussion

### Principal Results

Our study suggests that social media posts during the COVID-19 pandemic contain a significantly higher frequency of symptomatic mental health and support expressions than a comparable data set from the same period in the previous year. The effect sizes and statistical differences observed in our analyses provide evidence that COVID-19 may have led to mental health concerns compared to other normative times. We also found that they topically relate to the ongoing crisis situation and include concerns such as treatment, precautionary measures, loss of jobs, school closings, stockpiling of basic livelihood necessities, feeling lonely, boredom, and tired of the restrictions and constraints put on by the ongoing pandemic. Our findings suggest that although the COVID-19 pandemic has amplified mental health risks and concerns, it may have heightened a sense of belonging and solidarity among individuals—bringing them together, raising collective awareness, and encouraging them to provide support to one another. We also found expressions of solidarity with health care and high-risk workers, suggesting that people have been considerate about these workers and have expressed desire to set up opportunities for donating to those who have lost jobs during the crisis; this also aligns with recent media reports and World Health Organization guidelines of tackling the pandemic [77,78]. Media reports have also indicated how benevolent neighbors have been, tending to their older adult neighbors by delivering their groceries and other basic necessities [79].

However, mental health experts say that, although the crisis is amplifying risk factors for suicide, the COVID-19 outbreak’s effect on individuals’ mental and emotional well-being is complex [80]. Suicide is multifaceted, and although economic loss is a risk factor, so are depression, isolation, and fear of the future. At the same time, the crisis is possibly creating a sense of belonging for individuals at risk for suicide, as stress and anxiety are normalized, and people come together to better support one another during a crisis [81,82]. Our data showed a significant impact of COVID-19 on suicidal ideation, which calls for enhanced importance of population-scale mental health care, such as using approaches like universal screening (ie, Zero Suicide Model) [83]. As Florida [84] noted in a recent article, “The long-term toll on mental health of social isolation, remote work, and economic insecurity could have impacts akin to post-traumatic stress disorder; yet, the new focus on mental health may reduce stigma and increase the availability of support services.” Indeed, the world beyond the crisis may be one in which mental health is more honestly recognized and supported.

Interestingly, we noted that our findings indicate a gradual leveling out of these expressions, both symptomatic and supportive, which may reflect a developing *new normal*. In February 2020, it seemed unthinkable that the white-collar workforce of many countries would soon be working solely from home; it seemed unthinkable that air travel would plummet by 96% and that all major sporting events would be called off. Indeed, epidemiologists surmise that many if not most of the changes surrounding the rhythms of our daily life are likely to fade over time, just as they did after the 1918 influenza epidemic [84]. In other words, the pandemic could make us revisit and possibly reform many of our lifestyle choices and civic roles, and the persistent discussion of the *new normal* may help bring order to our current turbulence. Others have argued that perhaps the crisis is a prelude to a *new paradigm*, as recently noted by the World Economic Forum [84,85]: “Feeling unsettled, destabilized and alone can help us empathize with individuals who have faced systematic exclusions long-ignored by society even before the rise of COVID-19 – thus stimulating urgent action to improve their condition.” We should, therefore, “revel in the discomfort of the current moment to generate a ‘new paradigm,’ not a ‘new normal.’” The leveling out trend in our data gives empirical ground to these conjectures.

Nevertheless, if robust antiviral treatments are developed and rolled out relatively quickly or if a vaccine becomes available soon enough, presumably, the changes will be short-lived, and the new normal may be temporary. However, if the pandemic comes back in larger waves over the next few seasons, as was the case with historical epidemics, the economic, political, and social crises that have arisen as a consequence will lead to deeper ramifications in turn, leading to longer lasting or permanent changes. Future research will need to explore the persistence of the new normal and the emergence of a possible new paradigm as the pandemic evolves, and therein the mental health impacts further along in the crisis. A study like ours on the ongoing pandemic is a step toward leveraging large-scale online data to understand people’s response to the crisis and thinking about means to address the major concerns. Our study bears implications in digital technology driven mental health interventions to provide tailored support to people’s concerns during the crisis; a recent work by Rudd and Beidas [86] pointed out four point guidelines to build innovative and expansive solutions toward improving public mental health. The variety of concerns and help-seeking factors reflected in our study can also help several stakeholders, ranging across mental health facilitators and policy makers, toward early preparedness and interventions for mental health support. Similarly, our methods can offer the potential to build public health surveillance technologies that surface early warning signs of the effects of the various events related to the pandemic and other crises. The potential of social media to assist in the response to the pandemic is clear but also dependent on the accuracy of underlying methods. The reach of social media allows for broad access that transcends national borders or cultural differences. Using this access to meet the increasing need for help seeking, online and social media data is in a prime position to offer people personalized guidance toward accurate information, health care resources, and even basic lifestyle interventions. Underlying this potential is, thus, the ability of social media data to classify the state and needs of each individual and use that information to tailor a customized response. Precedents for such a system are abound as seen in several prior studies [27-31,37,87-89].

### Comparison With Prior Work

COVID-19 is not the first pandemic—catastrophic pandemics have been occurring at regular intervals throughout human history, with the 1918 influenza epidemic being the last one before the current pandemic [84,85,90]. The backdrop of the 1918 pandemic was that it happened just before the advent of modern psychiatry as a science and a clinical specialty—a time when psychoanalysis was gaining recognition as an established treatment within the medical community [91,92]. Consequently, psychiatry has had little opportunity to consider such historically important phenomena through its clinical, scientific lens until now. Although outbreaks of the Zika and Ebola viruses, Middle East respiratory syndrome, and severe acute respiratory syndrome managed to draw global attention, stirring up anxiety and uncertainty in societies, scholars have noted that participation of mental health experts in pandemic preparedness has remained negligible [93]. Consequently, our ability to understand mental health responses as well as the mental health burden in pandemic outbreaks have been limited [94]. For instance, a routinely practiced method of infection control, quarantine and social distancing, have received surprisingly little attention in psychiatric literature so far. Baumeister and Leary [95] contended that humans need frequent contacts, and crisis events further stimulate a need for affiliation and intimacy. Therefore, prolonged isolation and separation from families and their community can have profound effects on individuals even if they are not directly affected by the disease [4]. In the current pandemic, the additional layer of extensive social media use and exposure to often sensationalized online news while in physical isolation may add new complexities to implementing emotional epidemiology in managing concerns, fears, and misconceptions [96,97], as these tools have been argued to bear negative effects on psychological well-being [97,98].

By adopting social media as a lens to unpack these previously less understood dimensions of a pandemic’s mental health effects, our study is one step toward closing some of the previously noted gaps. The published literature posits that the distress and anxiety among individuals in this COVID-19 pandemic may increase the incidence of mental disorders [53,54,99]; data thus far from the United States point to a population increase in psychological distress of 10% compared to 2018 data [8], a trend that is in line with our results. These rates may be higher in those regions heavily exposed to COVID-19 or among individuals working during the pandemic, with a recent review reporting over 20% prevalence of anxiety, which is also consistent with our findings [8].

Prior work found that mental health discourse on Twitter ranges across stigmatizing, inspirational, resource, medical, and social dimensions of expressions [100], and our study revealed similar topical diversity in our data set. Further, we detected through social media many of the stresses associated with the pandemic (eg, prolonged isolation, exposure to pandemic-related death, loss of income and career, increased workload, and lack of pertinent and accurate information). These results align with epidemiological findings that COVID-19 has led to elevated mental health symptoms for individuals. Nelson et al [101] surveyed 2000 individuals from the United States, Canada, and Europe, and found elevated symptoms of anxiety and depression compared to historical norms and observed factors similar to the concerns we detected regarding symptomatic expressions and those related to seeking support. They also reinforced the summary data released by the Crisis Text Line (a major crisis helpline in the United States) listing major concerns of crisis support sought during this period [102], with 80% of conversations mentioning *“virus,”* 34% mentioning *“anxiety,”* 34% feeling solidarity with friends and family, etc. Along similar lines, there have been numerous reports about the increasing number of mental health crisis helpline calls during this period [103,104], providing further support and external validation that our social media findings reflect many of the same elements of distress expressed offline during this crisis.

Next, our temporal analyses pointed to a steady decline in people’s expressed psychosocial concerns during the 2-month study period (Figures 1 and 2), which conforms with similar findings in Google search queries as stay-at-home orders and other COVID-19–related policy changes were implemented in the United States [105]. We note contemporary social computing research studying various aspects of the social media discourse related to COVID-19 [63,106-108]. By providing complementary evidence to observations by Mackey et al [106] and Stokes et al [107] on expressed (mental health) concerns during the crisis, our study further underscores their findings using a comparable (control) data set, reinforcing and providing empirical credibility to the impression that the COVID-19 pandemic has indeed caused or contributed directly to the mental health concerns that we describe.

### Limitations and Future Work

We note some limitations in our study, many of which present excellent directions for future research. We recognize the lack of transparency related to the Twitter Streaming API. Recent research has also questioned the credibility of the “1% Twitter stream” aspect, noting that actual sampling data was smaller than what it ideally should have been [41]. Given these data limitations, we decided against conducting several descriptive and fine-grained analyses (such as comparing regions), and refrained from making claims based on comparing absolute numbers of those impacted by various mental health concerns. For example, we cannot define based on our data whether there were increased or decreased Twitter postings during our COVID-19 study period compared to the same months in 2019. Again, we chose to filter English-only Twitter posts given both algorithmic limitations of our methodologies and lower prevalence of non-English data (particularly in the US context). However, future work can extend our methodologies to conduct analyses in other languages to draw richer insights.

Despite the strengths of Twitter as a data source that provides us unobtrusive access to large-scale, unstructured, and naturalistic data of people’s candid self-disclosure and that it has been a valuable source to study disaster and crisis response [109], we acknowledge that this data inherently has many biases such as self-selection and representation [110]. We can only study those who self-select to express on Twitter. Pew Internet Center surveys reported that social media platforms are underrepresentative of minorities, although Twitter is an exception, which overrepresents minorities such as Blacks, Hispanics, and women [20]. There is already a digital divide in terms of social media use where the population is skewed toward young adults and white-collar workers. Further, technology and social media could be a luxury to marginalized and underprivileged populations, and any sort of technology-driven support and assistance will disproportionately affect different individuals based on technology use [88]. Similarly, a single platform cannot provide a complete picture; different platforms (eg, Facebook, Reddit, Twitter, and instant messaging services) have unique design strengths and weaknesses both in terms of their affordances as well as who uses them. Therefore, as highlighted in a recent article by Chunara and Cook [23], public health surveillance (including that for COVID-19) can account for several factors such as the “population at risk” in epidemiology and demographic disparities in the use and behavioral expressions on social media.

We understand that our study is observational and, as any other observational study, does not measure “true causality.” Watts [111] noted the impossibility to test all explanations and confounders simultaneously. However, by including and comparing against control data, we minimized geo-temporal and seasonal confounds, thereby enabling us to provide stronger evidence and insights than purely correlational analyses regarding the effects of the COVID-19 pandemic on people’s mental health. We also note that support expressions in our study can not only include support-seeking but also support-providing expressions. This has also enabled us to observe how solidarity and sense of belonging proliferated during the COVID-19 crisis. Future work can build separate high precision classifiers for each kind of expression to disentangle the prevalence of seeking and providing expressions during the crisis.

Further, although we did have data beyond May 24, 2020, we decided to exclude those to keep our focus on the effects on social media expressions due to COVID-19 and minimize those that followed the death of George Floyd on May 25, 2020, in the light of the Black Lives Matter protests throughout the United States [112]. We also are aware that, with the continuing nature of the pandemic, our conclusions are restricted to the mental health and support-seeking concerns expressed during a finite study period. Events since the end of the study period underscore the dynamic nature of these events, as different parts of the United States are heavily affected, while others are recovering, and some remain relatively spared. It will be important to extend this work temporally; increase the size of future samples; and, whenever possible, add geospatial specificity to future analyses. The latter will be especially important for potential supportive interventions locally if one has the resources and the ability to assemble recurring, near-real-time local “snapshots” as a basis for community-focused preventive interventions. Further, our analyses can be extended to retrospectively examine the aftereffects of particular global and local events, such as policy changes, related to the pandemic.

### Conclusion

Our study, like those of others studying other major events, further reinforces the potential utility of accessing and analyzing social media data in near-real-time to *take the temperature* of communities. This will require a more focused and robust collection of locally targeted information to build samples that are sufficiently large to produce reliably representative data sets to be useful for public health interventions. Further work is now needed to track mental health–related expressions and those reflecting needs for support throughout the pandemic, which has seen dynamic changes associated with disease spread to areas that had been less affected during the early months of the outbreak. This geo-specific research may further enhance our understanding of the causal connections between COVID-19 spread and waves of expressed distress. Having the ability to present locally pertinent, contemporaneous analyses offers the opportunity for local public health and mental health providers as well as political leaders to develop and deploy targeted support services in a timely fashion.

## Abbreviations

API: application programming interface
ES: emotional support
IS: informational support
LLR: log-likelihood ratio
SAGE: Sparse Additive Generative Model
SVM: support vector machine
